# Efficacy and safety of intravitreal injections of conbercept for the treatment of idiopathic choroidal neovascularization

**DOI:** 10.1186/s12886-024-03344-6

**Published:** 2024-02-19

**Authors:** Gaixia Zhai, Yuanzhen Su, Shaopeng Wang, Hui Lu, Na Liu

**Affiliations:** Zibo Central Hospital, Zibo, China

**Keywords:** Conbercept, Idiopathic choroidal neovascularization, Vascular endothelial growth factor, Efficacy, Safety, Multifocal electroretinogram

## Abstract

**Background:**

To determine the efficacy and safety of intravitreally injected conbercept, a vascular endothelial growth factor receptor fusion protein, for the treatment of idiopathic choroidal neovascularization (ICNV).

**Methods:**

This retrospective study analyzed outcomes in 40 patients (40 eyes) with ICNV who received intravitreal injections of conbercept 0.5 mg (0.05 ml) and were followed up for at least 12 months. All patients underwent full ophthalmic examinations, including best-corrected vision acuity (BCVA), intraocular pressure (IOP), slit-lamp examination, color fundus photography, optical coherence tomography angiography, multifocal electroretinogram, and fundus fluorescence angiography, if necessary, at baseline and after 1, 3, 6, and 12 months. BCVA, macular central retinal thickness (CRT), IOP, CNV blood flow area, thickness of the CNV-pigment epithelial detachment complex, thickness of the retinal nerve fiber layer (RNFL), and the first positive peak (P1) amplitude density in ring 1 before and after treatment were compared.

**Results:**

Mean baseline BCVA (logMAR), CRT, CNV blood flow area, and CNV-pigment epithelial detachment complex thickness were significantly lower 1, 3, 6, and 12 months after than before conbercept treatment (*P* < 0.05 each). IOP and baseline RNFL thickness were unaffected by conbercept treatment. P1 amplitude density was significantly higher 1, 3, 6, and 12 months after than before conbercept treatment (*P* < 0.05 each). None of the 40 eyes showed obvious ocular adverse reactions, such as endophthalmitis, glaucoma, cataract progression, and retinal detachment, and none of the patients experienced systemic adverse events, such as cardiovascular and cerebrovascular accidents.

**Conclusions:**

Intravitreal injection of conbercept is beneficial to eyes with ICNV, inducing the recovery of macular structure and function and improving BCVA, while not damaging the neuroretina. Intravitreal conbercept is safe and effective for the treatment of ICNV.

## Background

Choroidal neovascularization (CNV) is defined as the abnormal hyperplasia of a network of blood vessels from the choroidal vascular layer, extending beyond Bruch’s membrane to the retinal pigment epithelium (RPE) layer and/or the sensory layer [1, 2]. The causes of CNV include age-related macular degeneration, pathological myopia, angioid streaks, toxoplasmosis, and histoplasmosis of the eye [3]. Unexplained CNV in patients aged < 50 years is termed idiopathic CNV (ICNV) [4].

Vascular endothelial growth factor (VEGF) has been associated with the development of ICNV [5]. Similar to the treatment of wet age-related macular degeneration, anti-VEGF treatment is considered first-line therapy for patients with ICNV [6]. Conbercept is an anti-VEGF recombinant fusion protein developed in China that can block the binding of VEGF to its receptors and was approved in 2013 by the China Food and Drug Administration for the treatment of wet age-related macular degeneration [7]. Because VEGF has been associated with the development of CNV, conbercept may also inhibit the development of this condition.

Optical coherence tomography angiography (OCTA) is a non-invasive imaging technique that measures blood flow in the retina, enabling it to assess the morphology of the superficial, deep, and outer layers of the retina and choroidal capillary layers [8]. Electroretinography can detect changes in retinal function, and multifocal electroretinography (mfERG) can objectively and accurately detect and record changes in visual function in the 30° region of the posterior pole of the retina within a relatively short time. mfERG can also accurately locate regions of the retina with abnormal function, and can quantify changes in retinal function in the macular region [9].

During the embryonic development of the eyeball, VEGF plays key roles in the formation of blood vessels, in the proliferation of endothelial cells, and in the permeability of blood vessels [10]. VEGF can protect nerves, promote the generation of nerve cells, and inhibit the apoptosis and death of neurons [11]. Intravitreal injections of anti-VEGF drugs may can inhibit the normal protective effect of VEGF on nerves and cause neuroretinal damage. Few reports to date have described the effects of intravitreal injections of conbercept in patients with ICNV [12, 13].

The present study retrospectively analyzed the effects of intravitreal conbercept on 40 patients (40 eyes) with ICNV. To our knowledge, this present study is the first to utilize mfERG to monitor changes in macular function induced by intravitreal injections of conbercept in eyes with ICNV. The present study also assessed conbercept associated changes in retinal nerve fiber layer (RNFL) thickness to determine whether intravitreal injections of conbercept would have an effect on the neuroretina.

## Methods

The medical records of 40 patients (40 eyes) diagnosed with ICNV and treated with intravitreal injections of conbercept from January 2020 to June 2022 in Zibo Central Hospital were retrospectively assessed. Patients were included if they were aged < 50 years, provided written informed consent; underwent comprehensive ophthalmic examination and relevant imaging examinations; had sufficiently clear refractive interstitia for imaging tests; and if new lesions manifested within 1 month. Patients were excluded if their clinical data were incomplete; if they had ocular diseases, such as diabetic retinopathy, retinal vein obstruction, glaucoma, retinal detachment, pathologic myopia, age-related macular degeneration, toxoplasmosis, histoplasmosis of the eye, angioid streaks, or ocular hypertension; had previously undergone eye surgery or intravitreal injection; or had a refractive error ≥ 6.00D or an axial length ≥ 26.0 mm. This study adhered to the principles of the Helsinki Declaration and was approved by the Medical Ethics Committee of Zibo Central Hospital (NO.202001004).

All patients underwent relevant examinations, including measurements of best corrected visual acuity (BCVA), intraocular pressure (IOP), slit lamp examination, color fundus photography, optical coherence tomography-angiography (OCTA; Optovue Inc., Fremont, CA, USA), and mfERG, at baseline and 1, 3, 6, and 12 months after treatment. Reti-scan mfERG was performed using a multifocal ERG instrument, Version 3.15 (Roland Company, Germany). Fundus fluorescein angiography (FFA; Spectralis, Heidelberg, Germany) was deemed feasible if the number of CNV lesions had increased or new CNV lesions were suspected during follow-up. All patients received intravitreal injections of conbercept (0.05 mL/0.5 mg; Chengdu Kanghong Biotechnology Co., Ltd.) (1 + PRN) and were followed up for at least 12 months. Central retinal thickness (CRT), CNV- pigment epithelial detachment (CNV-PED) complex thickness, and CNV blood flow area were assessed using OCTA (Optovue RTVue xR Avanti, Optovue Inc. USA), with a scanning range of 3 × 3 mm for the macular area. CNV areas on the outer retina were manually selected by two qualified technicians using AngioAn-alytics software. Flow areas were automatically measured as the detected flow signals within the selected area of the CNV. RNFL thickness around the optic disc was measured using OCTA, with the scanning range around the optic disc being 4.5 × 4.5 mm. the first positive peak (P1) amplitude density (Amp-P1) in ring 1 was calculated based on mfERG. BCVA (LogMAR), IOP, CRT, CNV blood flow area, CNV-PED complex thickness, and RNFL thickness, and Amp-P1 before and after treatment was compared. The criteria for re-injection included new CNV lesions; increases in CNV blood flow area; reductionsin BCVA by more than one row; and > 50 μm increases in CRT.

During mfERG, the retina was graphically stimulated with a capture ray tube stimulator, reflecting the signal characteristics of various small parts of the retina. The recording electrode was a corneal contact lens electrode, and the stimulation pattern was composed of 61 black and white hexagons. The stimulation result was 5 rings, with half of the stimulation hexagons being black and the other half white. The colors of the black and white hexagon were randomly converted. After computer processing, the ERG waveform curve of the corresponding region of the retina could be obtained. The original waveform consisted of 61 individual waveforms, each composed of a negative N1 wave and a positive P1 wave. The Amp-P1 in ring 1 reflects the retinal function at the center of the macula [14].

All patients were hospitalized after the exclusion of systemic contraindications. Intravitreal injections were administered in a sterile operating room. Before surgery, the conjunctival sac was washed with povidone iodine, and conbercept was slowly injected into the vitreous cavity through a vertical needle 3.5–4 mm behind the lower temporal corneal limbus at the flat part of the ciliary body. After the surgery, levofloxacin eye drops were administered 4 times per day for 7 days.

All data are expressed as mean ± standard deviation (SD). The Anderson-Darling test was performed to determine whether the data were normally distributed. The independent sample t-test or Mann-Whitney test was used to compare the values at 1, 3, and 6 months after treatment with the last follow-up. The paired t-test or Wilcoxon signed-rank test was used to compare the values after treatment with those before treatment. All statistical analyses were performed using GraphPad Prism 9 statistical software, with *P* values < 0.05 indicating statistical difference.

## Results

The study included 40 eyes of 40 patients, 20 men and 20 women, of mean ± SD age 30.93 ± 4.99 years (range, 20 to 41 years). The mean ± SD time interval between symptom onset and start of therapy was 7.78 ± 3.79 days (Table 1).

**Table 1:** Basic demographic and ocular characteristics of included patients (*N* = 40)

Characteristics	Patients, *n* = 40 (eyes, *n* = 40)
Sex (eye)	
Male	20
Female	20
Age (years)	30.93 ± 4.99
Time from symptom onset to start of treatment (days)	7.78 ± 3.79
Type (eye)	
Subfoveal	40
IOP (mmHg)	15.45 ± 2.71

*IOP* intraocular pressure

**Fig. 1:**
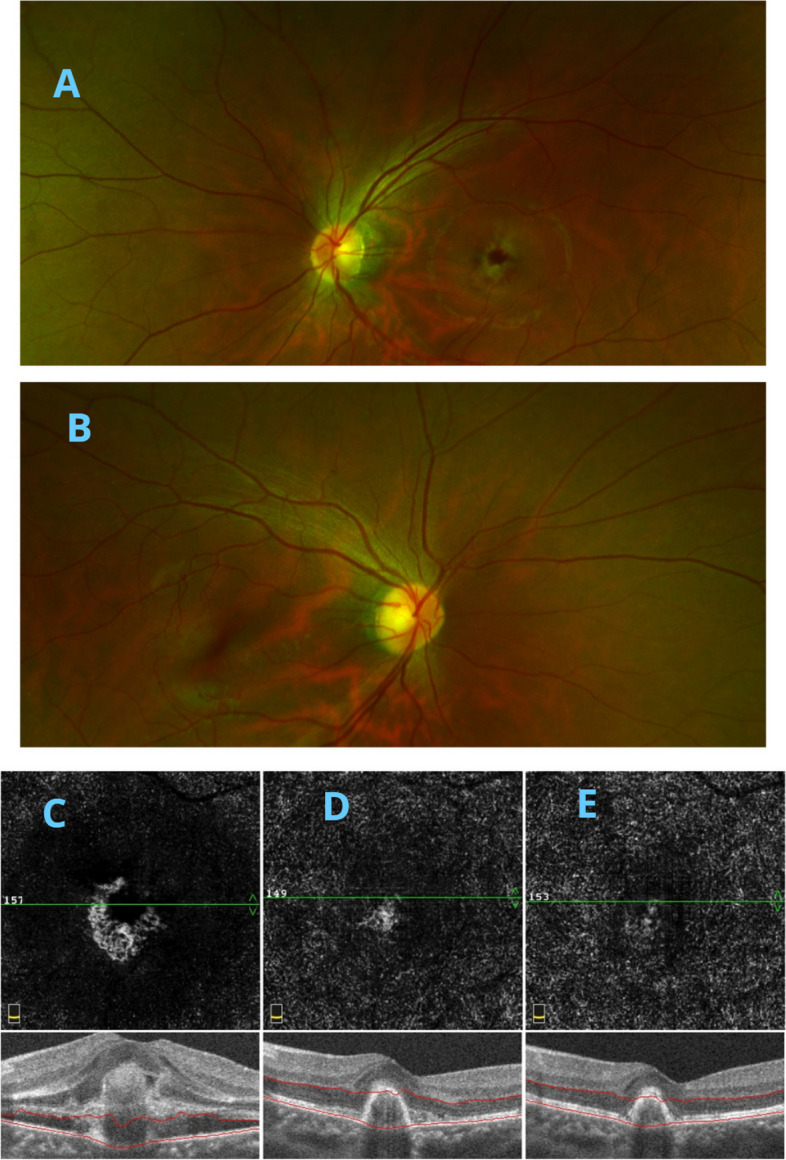
**A**,** B** Fundus photos of both eyes of a single patient before treatment. **C-E** OCTA results, showing that CNV blood flow area and CRT decreased significantly after treatment. OCTA, optical coherence tomography angiography; CRT, central retinal thickness

**Fig. 2:**
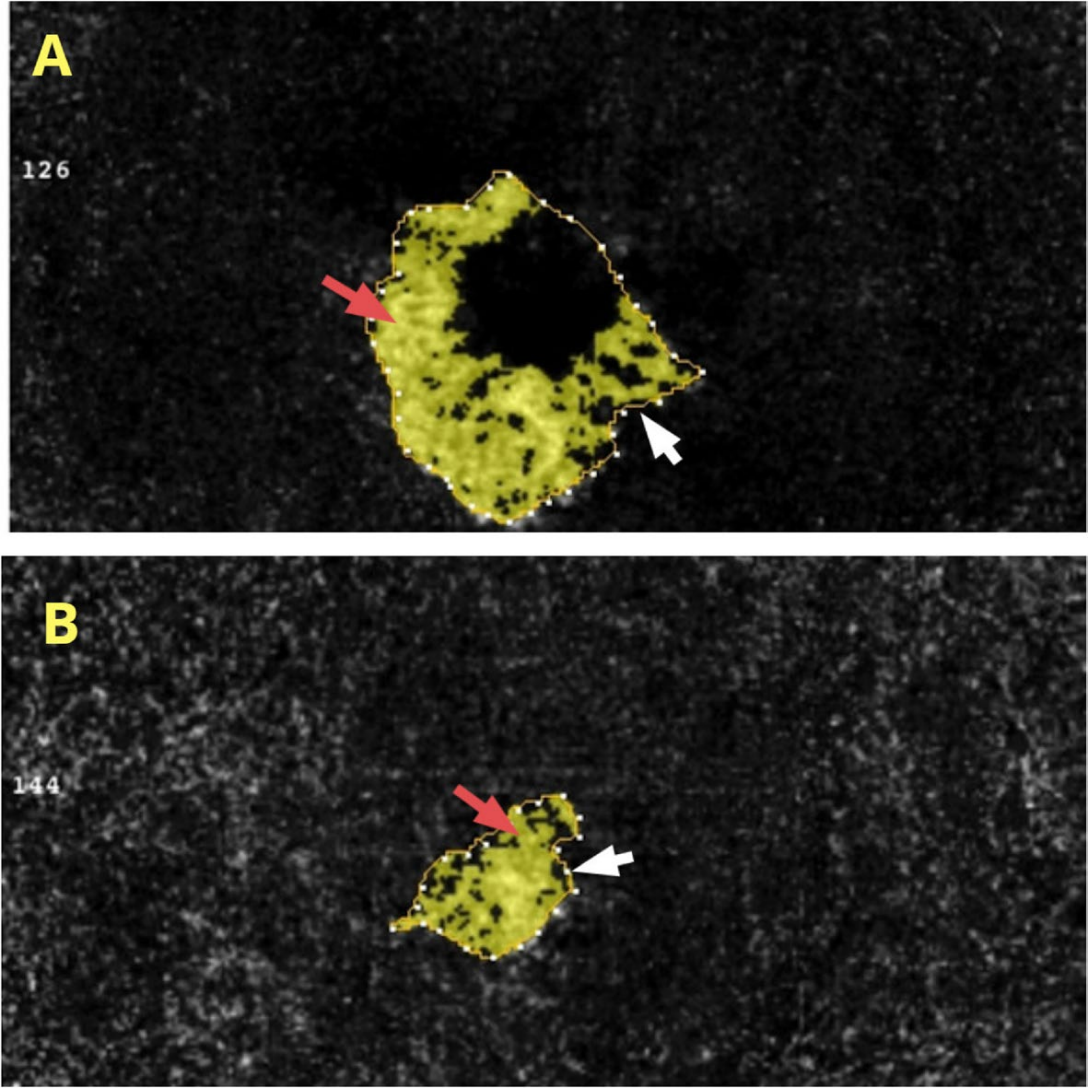
OCTA results, showing the CNV structures of the outer retinal layer (**A**) before and (**B**) after treatment. White arrows represent the selected CNV area and red arrows represent the CNV blood flow area. Abbreviations: OCTA, optical coherence tomography angiography; CNV, choroidal neovascularization

Mean ± SD BCVA was 0.45 ± 0.15 LogMAR at baseline, decreasing significantly to 0.28 ± 0.11, 0.24 ± 0.13, 0.24 ± 0.12, and 0.21 ± 0.10 LogMAR at 1,3, 6, and 12 months, respectively (*P* < 0.05) (Table 2). CRT also decreased significantly, from 314.2 ± 35.36 μm before treatment to 257.0 ± 30.35, 254.1 ± 31.28, 247.7 ± 29.24, and 251.1 ± 29.31 μm at 1, 3, 6, and 12 months, respectively (*P* < 0.05). The CNV blood flow area was 0.20 ± 0.05 mm^2^ before treatment, decreasing significantly to 0.13 ± 0.04, 0.12 ± 0.04, 0.13 ± 0.05, and 0.11 ± 0.03 mm^2^ at 1, 3, 6, and 12 months, respectively (*P* < 0.05). IOP was not significantly affected by conbercept treatment, being 15.45 ± 2.71 mmHg at baseline and 15.48 ± 2.25, 14.60 ± 2.48, 15.75 ± 2.24, and 14.88 ± 2.55 mmHg at 1, 3, 6, and 12 months, respectively (*P* > 0.05) (Table 2). The results of CRT and CNV area of one patient are shown in Fig. 1, and the CNV structure of the outer retinal layer is shown in Fig. 2.

**Table 2:** BCVA, CRT, CNV blood flow area, and IOP before and after treatment (*N* = 40)

Indices (mean ± SD)	Preoperative	1 month	3 months	6 months	12 months
BCVA (LogMAR)	0.45 ± 0.15	0.28 ± 0.11^* ^^	0.24 ± 0.13^* ^^	0.24 ± 0.12^* ^^	0.21 ± 0.10^*^
^*^p value	–	< 0.0001	< 0.0001	< 0.0001	< 0.0001
^^^*p* value	–	0.0033	0.2683	0.4451	–
CRT (μm)	314.2 ± 35.36	257.0 ± 30.35^* ^^	254.1 ± 31.28^* ^^	247.7 ± 29.24^* ^^	251.1 ± 29.31^*^
^*^p value	–	< 0.0001	< 0.0001	< 0.0001	< 0.0001
^^^*p* value		< 0.0001	0.6566	0.6103	–
CNV area (mm^2^)	0.20 ± 0.05	0.13 ± 0.04^* ^^	0.12 ± 0.04^* ^^	0.13 ± 0.05^* ^^	0.11 ± 0.03^*^
^*^p value	–	< 0.0001	< 0.0001	< 0.0001	< 0.0001
^^^*p* value		0.0361	0.7281	0.1057	–
IOP (mmHg)	15.45 ± 2.71	15.48 ± 2.25^# ^^	14.60 ± 2.48^# ^^	15.75 ± 2.24^# ^^	14.88 ± 2.55^#^
^#^*p* value	–	0.9632	0.1899	0.5911	0.2778
^^^*p* value		0.2686	0.6264	0.1073	–

*BCVA* best-corrected visual acuity, *logMAR* logarithm of minimal angle of resolution, *CRT* central retinal thickness, *CNV* choroidal neovascularization, *IOP* intraocular pressure. ^*^P < 0.05, ^#^*P* > 0.05 compared with the data before treatment. ^^^vs data at 12 months. The independent sample t-test or Mann-Whitney test was used to compare the values at 1, 3, and 6 months after treatment with the last follow-up. The paired t-test or Wilcoxon signed-rank test was used to compare the values after treatment with those before treatment

The CNV-PED complex thickness was reduced significantly, from 148.0 ± 13.15 μm at baseline to 119.7 ± 10.18, 95.68 ± 9.39, 89.75 ± 13.44, and 84.33 ± 10.75 μm at 1, 3, 6, and 12 months, respectively (*P* < 0.05) (Table 3). In contrast, mean RNFL thickness was not affected by conbercept treatment, being 105.0 ± 8.14 μm before treatment and 104.4 ± 7.89, 104.3 ± 6.98, 104.3 ± 7.63, and 104.8 ± 7.90 μm after 1, 3, 6, and 12 months, respectively (*P* > 0.05). Amp-P1, which was 49.08 ± 9.89 nv/deg^2^ at baseline, improved significantly, to 57.85 ± 9.65, 61.23 ± 9.38, 63.48 ± 8.96, and 64.65 ± 6.50 nv/deg^2^ at 1, 3, 6, and 12 months, respectively (*P* < 0.05) (Table 3).

**Table 3:** CNV-PED complex, RNFL, and Amp-P1 before and after treatment (*N* = 40)

Indices (mean ± SD)	Preoperative	1 month	3 months	6 months	12 months
CNV-PED complex (μm)	148.0 ± 13.15	119.7 ± 10.18^* ^^	95.68 ± 9.39^* ^^	89.75 ± 13.44^* ^^	84.33 ± 10.75^*^
^*^p value	–	< 0.0001	< 0.0001	< 0.0001	< 0.0001
^^^*p* value	–	< 0.0001	< 0.0001	0.0497	–
RNFL (μm)	105.0 ± 8.14	104.4 ± 7.89^# ^^	104.3 ± 6.98^# ^^	104.3 ± 7.63^# ^^	104.8 ± 7.90^#^
^#^*p* value	–	0.7977	0.0904	0.938	0.4489
^^^*p* value	–	0.7902	0.7650	0.7742	–
Amp-P1(nv/deg^2^)	49.08 ± 9.89	57.85 ± 9.65^* ^^	61.23 ± 9.38^* ^^	63.48 ± 8.96^* ^^	64.65 ± 6.50^*^
^*^p value	–	< 0.0001	< 0.0001	< 0.0001	< 0.0001
^^^*p* value	–	0.0017	0.0624	0.4501	–

*CNV-PED* choroidal neovascularization-pigment epithelial detachment, *RNFL* retinal nerve fiber layer, *Amp-P1* the first positive peak (P1) amplitude density. ^#^*P* > 0.05,^*^P < 0.05 compared with the data before treatment. ^^^vs data at 12 months. The independent sample t-test or Mann-Whitney test was used to compare the values at 1, 3, and 6 months after treatment with the last follow-up. The paired t-test or Wilcoxon signed-rank test was used to compare the values after treatment with those before treatment

Of the 40 treated eyes, 24 (60.0%) received one injection of conbercept, 11 (27.5%) received two injections, and five (12.5%) received three injections.

Corneal epithelial injury occurred in four eyes, with all showing epithelial repair after 3 days. Subconjunctival hemorrhage occurred in 7 eyes, with all hemorrhages absorbed after 7 days. One eye showed a transient increase in IOP, which was reduced to normal without any treatment. Four eyes experienced postoperative ocular hypotension. Of the 40 eyes, none experienced severe eye complications, such as endophthalmitis, cataract, glaucoma, or retinal detachment, and none of the 40 patients experienced cardio-cerebrovascular accidents.

## Discussion

Idiopathic choroidal neovascularization is a monocular disease that most often occurs in adults aged < 50 years [15, 16]. Similar to the pathogenesis of wAMD, the main cause of visual impairment in patients with ICNV is the formation of CNV in the macular area, followed by exudation, bleeding, and scarring [17]. Serum VEGF concentrations have been reported to be significantly higher in patients with ICNV than in a control group [17]. VEGF, the main promoter of neovascularization, binds to specific receptors on vascular endothelial cells, thereby increasing vascular permeability [18–20].

The pathogenesis of ICNV remains unclear [21, 22], and this condition cannot be treated based on its etiology. At present, the treatment of ICNV is similar to that of AMD, involving anti-VEGF therapy. The standard treatment plan for AMD involves intravitreal injections of an anti-VEGF agent once a month for 3 months, followed by once every other month on a pro re nata (PRN) basis [23]. Unlike AMD, CNV lesions are smaller in patients with ICNV than in patients with AMD, with the former characterized by mild edema and less bleeding. Patients with ICNV are usually treated using the 1 + PRN treatment plan [24–26]. Conbercept, a recombinant fusion protein, can block the binding of VEGF to its receptor and inhibit the formation of CNV. Conbercept has multiple targets, strong affinity to receptors, and a long duration of intraocular action [27].

The risks of intravitreal injections of anti-VEGF agents include cataract, glaucoma, intraocular hemorrhage, and endophthalmitis [28]. Although CNV progression can be effectively monitored by FFA, this method is invasive. In clinical practice, CNV progression and the need for treatment are determined primarily by spectral domain OCT (SD-OCT), which can assess subretinal, intraretinal, and subretinal fluids [29]. Alternatively, OCTA, a rapid, non-invasive method of monitoring blood flow, can quantitatively analyze blood flow and hierarchically monitor changes in chorioretinal blood flow changes [30]. OCTA can therefore quantitatively analyze CNV areas and predict responses to treatment, as well as to assess CNV shapes and guide clinical treatment.

Endogenous VEGF has protective and nutritional effects on nerve cells and plays an important role in the maintenance and function of adult retinal neurons [31]. Multiple intravitreal injections of conbercept inhibit endogenous VEGF and neovascular activity, which may have harmful effects on nerve cells. Studies assessing the effects of intravitreal injection of anti-VEGF drugs on RNFL thickness have yielded conflicting results, with some reporting that these drugs reduced RNFL thickness and others finding that these agents did not alter RNFL thickness [32, 33].

Multifocal electroretinogram, first described in 1992 [34], is a revolutionary development in visual electrophysiology. This method can also simultaneously record the local electrical responses of different small retinal areas and provide more relevant information for the clinical detection of retinal functional lesions.

The present study evaluated the efficacy and safety of conbercept for the treatment of ICNV by comparing BCVA, CRT, IOP, CNV blood flow area, CNV-PED complex thickness, RNFL thickness, and Amp-P1 before and after conbercept treatment. Several CNV diseases have similar clinical manifestations, however, making it difficult to diagnose patients based on auxiliary examinations. The present study therefore excluded patients with CNV who had high myopia, age-related macular degeneration, toxoplasmosis, angioid streak, tuberculin test positivity, and other clear causes of CNV, thus ensuring reliable patient enrollment. BCVA, CRT, CNV blood flow area, CNV-PED complex thickness, and Amp-P1 were found to differ significantly before and after conbercept treatment (P<0.05 each). Intravitreally injected conbercept was found to improve BCVA, reduce macular edema, reduce CNV lesions, and promote the recovery of macular structure and function. In contrast, conbercept treatment did not significantly alter IOP and RNFL thickness, indicating that intravitreal conbercept did not damage the neuroretina. The decrease in RNFL thickness around the optic disc after treatment may have resulted from reductions in retinal edema and overall retinal thickness after anti-VEGF treatment.

At the end of follow-up, 24 eyes (60.0%) had received one injection of conbercept, 11 (27.5%) had received two injections, and five (12.5%) had received three injections. This indicates that the 1 + PRN strategy may yield a good prognosis for most patients with CNV and reduce their financial burden.

One of the patients in this study, a 19-year-old woman, presented with low IOP and increased myopia after the intravitreal injections. Her IOP returned to normal, and her BCVA returned to preoperative levels after 3 days of treatment with cycloplegic agents. Incision leakage, ciliary body detachment, or choroidal detachment may occur after conbercept injections, but this may be related to the ocular structural characteristics of the patients. No serious ocular complications, such as endophthalmitis, cataract, glaucoma, or retinal detachment, or systemic complications, such as cardiovascular or cerebrovascular accidents, were observed.

This study had several limitations, including its small sample size and short duration. Long-term studies in larger numbers of patients are warranted.

## Conclusions

Intravitreal injections of conbercept for the treatment of ICNV may promote the recovery of structure and function in the macular region and improve BCVA, without causing damage to the neuroretina. Intravitreal conbercept is safe and effective for the treatment of patients with ICNV.

## Data Availability

The datasets generated and/or analyzed during the current study are not publicly available due to the prevision of further publications coming in the future but are available from the corresponding author on reasonable request.

## Abbreviations

BCVA: Best-corrected visual acuity
CRT: Central retinal thickness
CNV: Choroidal neovascularization
IOP: Intraocular pressure
CNV-PED: Choroidal neovascularization-pigment epithelial detachment
FFA: Fundus fluorescein angiography
OCTA: Optical coherence tomography angiography
PRN: Pro re nata
RNFL: Retinal nerve fiber layer
SD: Standard deviation
VEGF: Vascular endothelial growth factor.
